# Impact of the Uridine–Cytidine Kinase Like-1 Protein and IL28B rs12979860 and rs8099917 SNPs on the Development of Hepatocellular Carcinoma in Cirrhotic Chronic Hepatitis C Patients—A Pilot Study

**DOI:** 10.3390/medicina54050067

**Published:** 2018-09-27

**Authors:** Arida Buivydiene, Valentina Liakina, Elena Kashuba, Jolita Norkuniene, Skirmante Jokubauskiene, Egle Gineikiene, Jonas Valantinas

**Affiliations:** 1 Center of Hepatology, Gastroenterology and Dietetics, Clinic of Gastroenterology, Nephrourology and Surgery, Vilnius University, LT-08661 Vilnius, Lithuania; arida.buivydiene@santa.lt (A.B.); jonas.valantinas@santa.lt (J.V.); 2 Department of Chemistry and Bioengineering, Faculty of Fundamental Sciences, Vilnius Gediminas Technical University, LT-10223 Vilnius, Lithuania; 3 Department of Microbiology, Tumor and Cell Biology, Karolinska Institutet, SE-171 77 Stockholm, Sweden; elena.kashuba@ki.se; 4 RE Kavetsky Institute of Experimental Pathology, Oncology and Radiobiology, UA-03022 Kyiv, Ukraine; 5 Department of Mathematical Statistics, Faculty of Fundamental Sciences, Vilnius Gediminas Technical University, LT-10223 Vilnius, Lithuania; j.norkuniene@hotmail.com; 6 Vilniaus Kolegija/University of Applied Sciences, LT-08105 Vilnius, Lithuania; 7 Department of Pathology, Forensic Medicine and Pharmacology, Faculty of Medicine, Vilnius University, LT-03101 Vilnius, Lithuanian, skirmante.jokubauskiene@mf.vu.lt; 8 National Center of Pathology, LT-08406 Vilnius, Lithuania; 9 Center of Hematology, Oncology and Transfusion Medicine, Vilnius University Hospital Santaros Klinikos, LT-08661 Vilnius, Lithuania; egle.gineikiene@santa.lt; 10 Life Sciences Center, Vilnius University, LT-10257 Vilnius, Lithuania

**Keywords:** hepatitis C, liver cirrhosis, hepatocellular carcinoma, uridine–cytidine kinase like-1, interleukin 28B

## Abstract

*Background and objectives*: The hepatitis C virus (HCV) is the major causative agent of hepatocellular carcinoma (HCC) in the western world. The efficacy of surveillance programs for early detection of HCC is not satisfactory: many tumors are diagnosed at the late, incurable stages. Therefore, there is a need in reliable prognostic markers for the proper follow-up of HCV-positive patients. The aim of the present study was to assess the prognostic value of the uridine–cytidine kinase-like protein 1 (UCKL-1), a putative oncoprotein, together with genetically determined polymorphisms in the interleukin 28B (IL28B) gene (rs12979860, rs8099917) in the development of HCC in HCV-positive cirrhotic patients. *Materials and Methods*: We included 32 HCV cirrhotic patients, 21 (65.6%) of whom had HCC. The expression of UCKL-1 was assessed in liver tissue sections, using immunohistochemistry. For IL28B rs12979860 and rs8099917 genotype analysis, the corresponding genomic regions were amplified by polymerase chain reaction (PCR) with appropriate primers. *Results:* We have found that UCKL-1 expression was significantly increased in HCC (*p* = 0.003). The presence of rs8099917 TT single-nucleotide polymorphism (SNP) elevated the chances of HCC manifestation more than sevenfold (OR = 7.3, *p* = 0.0273). The presence of rs12979860 CC SNP also heightened HCC chances more than sevenfold (OR = 7.5, *p* = 0.0765). Moreover, in the HCC group, a combination of IL28B rs12979860 non-TT and rs8099917 TT genotypes was observed more often, compared with the non-HCC group. Other combinations of IL28B rs12979860 and rs8099917 SNIPs were associated with a reduced risk of HCC development, approximately at the same extent. *Conclusions*: The presence of IL28B rs8099917 TT and rs12979860 CC SNPs, but not the intensity of UCKL-1 expression, is strongly associated with increased chances of HCC development in HCV-positive cirrhotic patients.

## 1. Introduction

The burden of mortality due to liver cancer is in second place after lung cancer worldwide [1]. Hepatocellular carcinoma (HCC) represents more than 90% of primary liver cancers [2]. A major risk factor for HCC development is liver cirrhosis; about 80–90% of detected HCC cases are found in cirrhotic liver [3]. Once cirrhosis is developed, the annual risk of HCC development is 1–5% [4]. Unfortunately, both cirrhosis and HCC have a prolonged subclinical silent period. HCC is usually diagnosed at the late incurable stages or during surveillance for HCC in cirrhosis using ultrasound examination every six months, according to the guidelines from the European Association for the Study of the Liver (EASL), the European Organization for Research and Treatment of Cancer (EORTC), and the American Association for Study of Liver Disease (AASLD) [2,5]. When HCC already presents symptoms, the cure is practically impossible [6]. However, if HCC is discovered at an early stage, it is still curable [7,8].

The number of HCC incidences in the Nordic region, which includes Lithuania, is up to 10/100,000 inhabitants [9]. Noteworthy, in more than one-third of HCC cases, chronic infection with HCV is found [10]. In Lithuania, anti-HCV prevalence in adults (age under 18) was about 2.78% in 2010 [11].

Antiviral therapy can reduce but not completely eliminate the risk of developing HCC in patients with chronic hepatitis C (CHC) [12,13,14]. The molecular mechanisms of HCV-induced development of HCC are not fully characterized. It was proposed that the main role of HCV in carcinogenesis is a creation of the specific microenvironment in the cirrhotic liver tissue that enables carcinogenesis [15]. The uncontrolled cell proliferation and avoided apoptosis are due to accumulated genetic and epigenetic changes, leading to activation of oncogenes, hypersecretion, reduced tumor suppressor gene expression, and disorder in cellular signaling. The result of these alterations is malignant cell transformation.

In order to improve the selection of patients with HCV-associated cirrhosis and at higher risk for HCC development, different sets of potentially pro-carcinogenic and carcinogenesis-reflecting factors are under investigation worldwide [5,16]. Proteins involved in the control of cell cycle and apoptosis regulation can be used as tumor markers, also for HCC. For example, functionally disrupted p53 [17], retinoblastoma protein [18], β-catenin [19], and many others can be considered as tumor markers in the certain conditions.

UCKL1 (NP_060329) is a protein that might have a catalytic activity as uridine–cytidine kinase and as phosphoribosyltransferase. Such activity was attributed to a salvage pathway, i.e., the synthesis of RNA from the products of degradation. The uridine kinase is involved in an enhanced synthesis of RNA, i.e., it is upregulated in cancerous cells and during wound healing. It was suggested that nuclear localization of UCKL1 might be followed by nuclear UTP accumulation that speeds up RNA synthesis, which is needed for the rapidly proliferating cells during blast transformation [20].

It is already known that the catalytic activity of uridine kinases is increased 5–13-fold in various human tumors, for example, colon [21], ovarian, and liver [22]. Also, its activity is quite high in damaged tissues [23].

Importantly, upon downregulation of UCKL1 by siRNA, cells proliferate slower and undergo apoptosis [24]. Also, UCKL1-depleted cells are more prone to NK-cell recognition [24]. This suggests that UCKL-1 plays an important role in tumor cell survival.

In order to monitor the expression levels of the UCKL1 protein in HCC, immunohistochemistry on tumor samples was performed. The upregulation of the catalytic activity allows us to predict that this protein might be considered a prognostic marker.

Epidemiologic factors also make a significant impact on the increasing risk of development of HCC associated with HCV. For example, the aging of patients with HCV is associated with an increasing risk of HCC [7,25].

IL28B (more often named IFNL3) belongs to a type III family of interferons, namely IFNλ, which includes IFNλ1, IFNλ2, and IFNλ3. As a representative of the type III interferons, IL28B collaborates with the type I interferons (IFNα/β) in combating viral infections by downregulation of the TH2 response and upregulation of the TH1 response, as well as by induction of tolerogenic dendritic cells (DCs) and consequent promotion and expansion of Treg cells [26]. Hepatocytes demonstrate high IFN-λR1 gene expression rates; that is why IFNλ could be considered an important player in the eradication of HCV infection [27,28,29]. Moreover, the association between SNPs of the IL28B gene and the outcome of HCV infection was already described [26]. Thereafter, extensive data were published on this topic, especially on the influence of the polymorphism of IL28B SNPs on spontaneous HCV clearance and on the prognosis of the antiviral outcome [6,30,31,32,33].

The rs12979860 polymorphism, with its particular localization upstream of the promoter region of the IL28B gene as well as its proximity to the IFNL1 and IFNL2 genes, can theoretically influence all three IFNλ genes [34]. Clearly, polymorphism of rs12979860 T/C and SNPs in other regions (rs8099917 and the newly established rs368234815) of the IL28B gene do affect the expression of IFNλ3 in the liver and peripheral blood mononuclear cells, resulting in lower IFNλ3 expression [35].

Recent data showed that polymorphism in the upstream IFNL4 gene, besides that in the IL28B (IFNλ3) gene, influences necro-inflammation severity and liver fibrosis progression in CHC patients [36,37].

On the other hand, there are data providing clear evidence of an IFNλ inhibitory effect on the proliferation of various tumor cells, including HCC cells [38,39]. Therefore, we wanted to investigate the pattern of distribution of genetically determined polymorphisms of the IL28B gene (rs12979860, rs8099917) among individuals in our cohort, including HCC-bearing and tumor-free patients.

In the presented study, we aimed to elucidate how expression of the putative oncoprotein UCKL-1 in the liver together with IL28B polymorphism might be associated with HCC manifestation and progression in cirrhotic CHC patients, with the expectation of a synergistic effect between these markers in HCC pathology.

## 2. Materials and Methods

### 2.1. Patient Cohort

The present study was approved by a Vilnius Regional Biomedical Research Ethics Committee (permission No.158200-13-698-224). All patients signed an informed consent form before inclusion. A retrospective study was performed on tissues of 32 HCV cirrhotic patients that were treated at Vilnius University Hospital Santaros Klinikos during 2013–2016. The patients were divided into HCC and non-HCC groups, that included 21 and 11 patients, respectively. The HCV cirrhotic patients were aged from 32 to 70 years; 21 patients (65.6%) had HCC. Demographic variables (age, gender, body mass index), histological findings, as well as HCV data were assessed for all the patients (Table 1). In parallel with UCKL-1 expression, the IL28B gene polymorphism was investigated (rs12979860 and rs8099917).

### 2.2. Analysis of UCKL-1 Expression in the Liver Tissue

Liver histological samples and operative material obtained by hepatic resection or liver transplantation were fixed in a 10% buffered formalin solution. Immunohistochemistry (IHC) with anti-UCKL-1 antibody was performed on tissue microarrays (TMAs). The TMAs were constructed from 10% buffered formalin-fixed paraffin-embedded tissue blocks, selected by the pathologist. Cores of one millimeter in diameter were punched from selected areas. Paraffin sections of the TMAs were cut (2 µm thick), de-paraffinized, and re-hydrated, followed by heat-induced antigen retrieval in EnVision FLEX target retrieval solution at low pH for 20 min and cooling at room temperature for 15 min. The assay was performed using an automatic staining Link instrument (DAKO; Agilent Technologies, Inc., Santa Clara, CA, USA). The two-step IHC procedure was used with the EnVision FLEX detection system procedure (DAKO). The primary antibody against UCKL-1 (Sigma-Aldrich; St. Louis, MO, USA) was applied for 60 min (dilution 1:200), and the secondary antibody FLEX/HRP (DAKO) for 20 min. The peroxidase enzyme was then localized with 3,3′-diaminobenzidine, tetrahydrochloride, and hydrogen peroxide. As a counter stain, a hematoxylin solution was applied for 10 min. Visual evaluation of UCKL-1 was performed by the pathologist. Each spot was graded individually. UCKL-1 cytoplasmic reaction was evaluated as negative (when positive cells were absent)—0 reaction intensity points, weak (+)—1 point, moderate (++)—2 points, and strong (+++)—3 reaction intensity points; also, the percentage of cells with positive reaction was estimated visually in every sample. The relative units of UCKL-1 expression were calculated by multiplying of cytoplasmic reaction intensity points and percentage of positive cells.

### 2.3. Detection of Single-Nucleotide Polymorphism in IL-28 rs12979860 and rs8099917

Genomic DNA was extracted from blood samples, using the QIAmp DNA Blood Mini QIAcube Kit (Qiagen; Hilden, Germany), according to the manufacturer’s instructions. For IL28B rs12979860 and rs8099917 genotype analysis, corresponding genomic regions were amplified, using the following primers: for rs12979860—forward 5‘-GCGC TTAT CGCA TACG GCTA-3′ and reverse 5′-TATG TCAG CGCC CACA ATTC-3′; and for rs8099917—forward 5′-GTTC CTTG TAAA AGAT TCCA TCCA-3′ and reverse 5′-CAA CCC CAC CTC AAA TTA TCC-3′. Amplifications were performed at standard PCR conditions: 100 ng genomic DNA, 10 µL Maxima HotStart PCR Master Mix (2X) (Thermo Fisher Scientific; Waltham, MA, USA), and 0.2 µM of forward and reverse primers in a final volume of 20 µL. The cycling conditions were as follows: 2 min at 50 °C followed by 5 min at 95 °C and 40 amplification cycles at 95 °C for 15 s, 58 °C for 30 s, and 72 °C for 30 s.

Following amplification, the PCR products were sequenced with amplification primers using BigDye^®^ Terminator v1.1 Cycle Sequencing Kit (Applied Biosystems; Foster City, CA, USA). The sequencing reactions were analyzed by standard capillary electrophoresis, using Applied Biosystems 3500 series DNA Analyzer (Carlsbad, CA, USA) and VectorNTI v.11 Software (Thermo Fisher Scientific).

### 2.4. Statistical Analysis

Statistical analysis was performed using R-3.4.3 for Windows (GNU project). The Kolmogorov–Smirnov test was used to assess the data normality. Group differences were determined, using the Student t-test, when data distribution was normal; in other cases, the Mann Whitney and the Kruskal–Wallis criteria were used. The χ^2^ tests were conducted for categorical variables. The logistic regression model was used to investigate the association between age and gender of patients, biochemical test results and histological indices, HCV genotypes and viral load, intensity of the UCKL-1 expression in the liver tissue, IL28B rs12979860 and rs8099917 polymorphism and HCC development. All results were considered significant when α ≤ 0.05.

## 3. Results

Demographic, clinical, and laboratory parameters are shown in Table 1. Most patients in the non-HCC group were of 40–50 years old, while they patients were somewhat older (50–60 years old) in the HCC group. The gender distribution in both groups did not differ significantly (*p* = 0.434).

Body mass index (BMI) ranged from 18.8 to 34.9 (25.96 ± 4.19) and did not differ in the groups either (*p* = 0.706).

The genotype distribution in the studied cohort was as follows: HCV genotype 1 (GT1), 21 out of 32 patients (65.6%); genotype 3 (GT3), 9 out of 32 patients (28.1%); genotype 2 (GT2), only 2 out of 32 patients (6.3%). The HCV genotypes did not differ in HCC and non-HCC groups (*p* = 0.307).

The majority of patients in the HCC and non-HCC groups (23 out of 32 patients (71.9%) underwent treatment with pegylated interferon and ribavirin before development of HCC. In two subjects, HCC developed after HCV cure.

The statistical analysis did not confirm the difference of IL28B rs12979860 SNPs distribution in both groups (*p* = 0.481); however, in the HCC group, non-TT cases were presented more often. A significant difference in the distribution of IL28B rs8099917 SNPs between the studied groups was found (*p* = 0.015). In the HCC group, the IL28B rs8099917 TT genotype was observed more often than the non-TT genotypes (61.9% vs. 18.2%, *p* < 0.05, respectively) (Table 2).

This finding was confirmed by univariate logistic regression analysis (Table 3). The presence of rs8099917 TT SNP increases the chances of HCC manifestation more than sevenfold (OR = 7.3, *p* = 0.0273). The presence of rs12979860 CC SNP also increases HCC chances more than sevenfold (OR = 7.5, *p* = 0.0765). However, the result was not significant at the significance level *p* ≤ 0.05.

**Figure 1:**
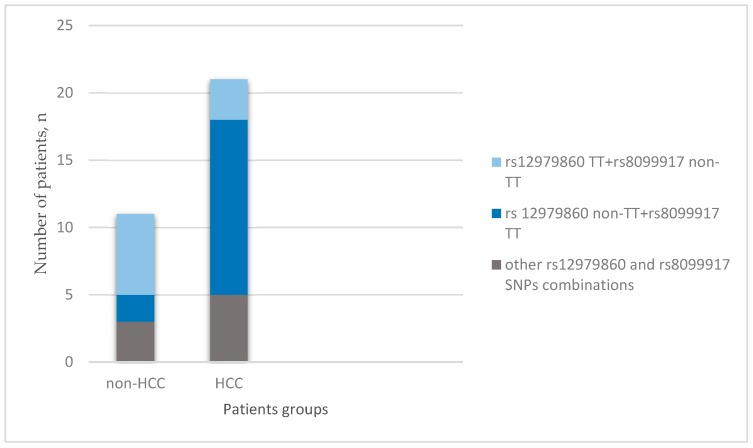
IL28B rs12979860 and IL28B rs8099917 single-nucleotide polymorphism (SNP) combinations in the HCC and non-HCC groups.

Moreover, in the HCC group, the combination of the IL28B rs12979860 non-TT and the rs8099917 TT genotypes was observed more often compared to the non-HCC group (Figure 1).

Other IL28B rs12979860 and rs8099917 SNIPs were associated with a reduced risk of HCC development (Table 3). According to the univariate logistic regression analysis, the presence of IL28B rs8099917 TG SNP reduces the chances of HCC more than fivefold, and IL28B rs12979860 TT SNP more than sevenfold (OR = 0.1786, *p* = 0.0333 and OR = 0.1389, *p* = 0.0231, respectively).

Noteworthy, UCKL-1 expression in liver tissue was found stronger in HCC (*p* = 0.003) (Table 1). However, according to the data of univariate logistic regression, this elevation was only slightly associated with the risk of HCC development (OR = 1.0279, *p* = 0.01) (Table 3). The same is true for the age of patients (OR = 1.1882, *p* = 0.0326).

None of the tested variables (patient age and gender, biochemical and histological parameters of liver injury, HCV genotypes and viral load, UCKL-1 expression level, IL28B SNPs variants) was confirmed as an independent predictor of HCC development in multivariate logistic regression analysis.

## 4. Discussion

The HCV genotype in the study groups did not differ from that in the general population: the most prevalent genotype was GT1, and the second most common genotype was GT3, regardless of the presence or absence of HCC [40,41,42]. HCC development in only two patients who were cured of HCV confirms the well-known phenomenon that a successful antiviral therapy in cirrhotic patients can reduce but not completely eliminate the risk of HCC development [12,13,14].

Contrary to the published data [30,43], the TT genotype for the rs8099917 of IL28B was found more often than the non-TT genotypes (61.9% vs. 18.2%, *p* = 0.015) and, moreover, it was confirmed as a HCC predictor (OR = 7.3125, *p* = 0.0273).

We could not confirm the impact of the IL28B rs12979860 TC genotype on HCC development (*p* = 0.481), in contrast to other studies [30,43,44]; however, the CC genotype was found associated with HCC development although without statistical significance at level *p* ≤ 0.05, most likely due to the small cohort size. (OR = 7.5, *p* = 0.0765). Our data are in concordance with earlier findings showing that IL28B SNPs alone do not increase the risk for HCC development; it is necessary to assess both their mutual combinations and combinations with other carcinogenic factors, for example, old age of patients [45]. The IL28B rs12979860 CC or TC polymorphisms in combination with the rs8099917 TT polymorphisms were found more often in the HCC group than in the non-HCC group. Also, there was no established protective effect of the IL28B rs12979860 CC polymorphism against HCC, as was suggested by other groups [46,47].

Since UCKL-1 was implicated in enhanced cell proliferation, as was mentioned earlier [20,48], and it could be involved in carcinogenesis, we expected a remarkable difference in its expression in cases of HCC compared with HCC-free liver cirrhosis patients. Indeed, UCKL-1 expression was more than twice higher in liver samples from HCC patients. However, according to univariate logistic regression analysis, the higher UCKL-1 expression does not significantly rise the odds of HCC development (OR = 1.0278587, *p* = 0.01). Summarizing, the reduced IFNλ production in hepatocytes as a result of SNPs in the responsible gene is more important for HCC development than the increase of UCKL-1. Of course, this premise should be proven by further studies.

## 5. Study Limitations

The main limitation of the presented study is the cohort size. In addition, in parallel to protein expression, UCKL-1 gene expression should be studied.

The data elucidating IL28B SNPs influence on HCC development obtained in our study are in concordance with data of Japanese researchers but contradict other published studies. This issue should also be considered carefully and requires more detailed investigation.

Prospective studies with a larger number of patients with HCV cirrhosis should be conducted to validate our findings.

## 6. Conclusions

The presence of IL28B rs8099917 TT and rs12979860 CC SNPs but not the intensity of the UCKL-1 expression is associated with increased odds of HCC development in HCV-positive cirrhotic patients.

## Figures and Tables

**Table 1:** Demographic, clinical, and laboratory characteristics in hepatocellular carcinoma (HCC) and non-HCC groups.

Characteristics	HCC (*n* = 21)	Non-HCC (*n* = 11)	*p* Value
Age, mean ± SD, years	55.14 ± 5.46	48.36 ± 8.96	0.012
Gender, *n* (%):			
Women	4 (19)	4 (36.4)	0.434
Men	17 (81)	7 (63.6)	
BMI, mean ± SD, kg/m^2^	25.75 ± 4.29	26.36 ± 4.15	0.706
HCV genotype, *n* (%):			
G1	12 (57.1)	9 (81.8)	0.307
G2	2 (9.5)	0 (0)	
G3	7 (33.3)	2 (18.2)	
Hepatitis C treatment, *n* (%)	14 (66.7)	9 (81.8)	0.506
HAI, mean ± SD, grade	6.52 ±1.86	6.64 ±2.34	0.883
Steatosis, mean ± SD, %	13.8 ± 17.1	7.7 ± 9.8	0.287
UCKL-1, mean ± SD, relative units	105 ± 58.52	43.18 ± 31.01	0.003

SD—standard deviation; BMI—body mass index; HCV—hepatitis C virus; HAI—histological activity index; UCKL-1—uridine–cytidine kinase-like protein 1

**Table 2:** IL28B gene polymorphisms in HCC and non-HCC groups.

IL28B Polymorphisms	HCC (*n* = 21)	Non-HCC (*n* = 11)	*p* Value
rs12979860, *n* (%):			
CC	9 (42.9)	1 (9.1)	0.481
TC	9 (42.9)	4 (36.4)	
TT	3 (14.3)	6 (54.5)	
rs8099917, *n* (%):			
TT	13 (61.9)	2 (18.2)	0.015
TG	5 (23.8)	7 (63.6)	
GG	3 (14.3)	2 (18.2)	

IL28B—interleukin 28B.

**Table 3:** Results of binary univariable logistic regression analysis for HCC predictors.

Variable	OR	95% Confidence Interval	*p*
UCKL-1	1.0279	1.0066–1.0496	0.0100
IL28B rs8099917 TT	7.3125	1.2490–42.8134	0.0273
IL28B rs8099917 TG	0.1786	0.0365–0.8728	0.0333
IL28B rs12979860 TT	0.1389	0.0253–0.7631	0.0231
IL28B rs12979860 CC	7.5000	0.8067–69.7326	0.0765
Age	1.1882	1.0144–1.3919	0.0326
